# Supplementary material for: AlphaFold 3 accurately models natural variants of Helicobacter pylori catalase KatA
Source: Microbiol Spectr. 2025 Aug 12;13(9):e00670-25. doi: 10.1128/spectrum.00670-25 (PMC12403698; doi:10.1128/spectrum.00670-25)
Supplement: Supplemental figure and table — Figure S1 and Table S1. [file spectrum.00670-25-s0001.docx]

**Supplementary material**

**
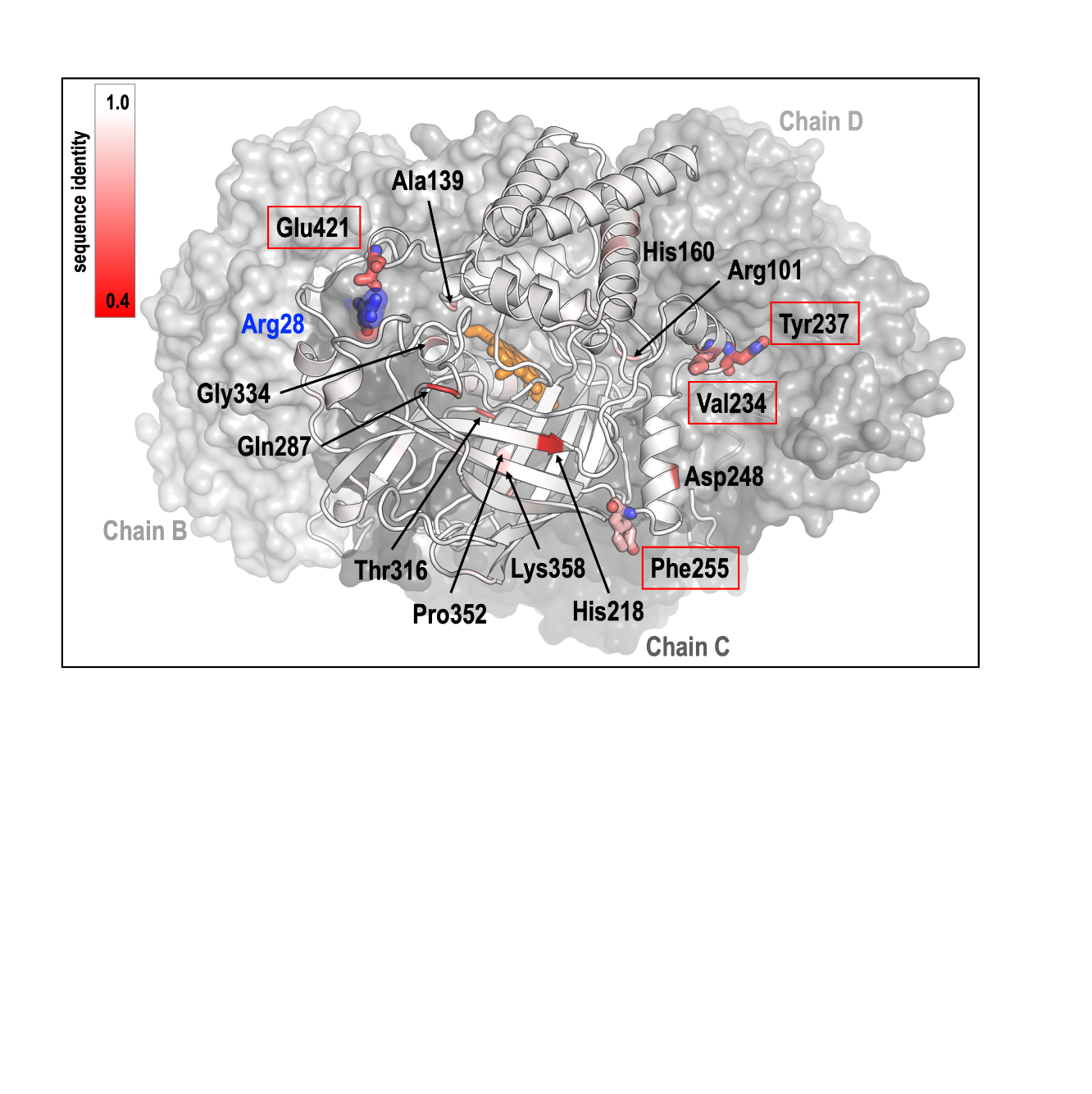
**

Fig. S1. Amino acid conservation mapped onto the *H. pylori* KatA_SS1_ structure.
The biologically relevant KatA tetramer is shown with Chains A-D colored in white, light gray, black, and dark gray, respectively. Sequence identity (see Data S1) at each position is mapped onto Chain A using a red-white color scale (0.4–1.0); red indicates these positions exhibit high variation in amino acid identity. Positions with sequence identity below 0.80 are labeled according to the KatA_SS1_ sequence. The four variant residues of interest in this study (Val234, Tyr237, Phe255, and Glu421) are shown as sticks and highlighted with red boxes. Arg28 of Chain B is noted in blue, which makes a key interaction with Glu421 in Chain A.

| **Table S1.** Summary of crystallographic statistics. | | |  | | |  |  |
| --- | --- | --- | --- | --- | --- | --- | --- |
| Protein, PDB code | *H. pylori* KatA_SS1_, PDB: 9NH3 | |  |  |  |  |  |
| Space Group | *P* 2_1_ 2 2_1_ | |  |  |  |  |  |
| Cell dimensions and angle  (a, b, c, α, β, γ) (Å, º) | 67.5, 96.2, 154.8, 90, 90, 90 | |  |  |  |  |  |
| Resolution (Å) ^a^ | 55.3-1.87 (1.94-1.87) | |  |  |  |  |  |
| Completeness (%) ^a^ | 100.0 (100.0) | |  |  |  |  |  |
| Total reflections | 1087793 (107235) | |  |  |  |  |  |
| Unique reflections | 84023 (8292) | |  |  |  |  |  |
| Average *I/σ* ^a^ | 8.45 (1.50) | |  |  |  |  |  |
| R_merge_ ^a^ | 0.28 (1.42) | |  |  |  |  |  |
| CC_1/2_ ^a^ | 0.99 (0.57) | |  |  |  |  |  |
| R_work_ (%) | 14.3 | |  |  |  |  |  |
| R_free_ (%) | 19.0 | |  |  |  |  |  |
| Ramachandran favored, allowed, outliers (%) | 96.0, 4.0, 0.0 | |  |  |  |  |  |
| Non-hydrogen atoms | 9435 | |  |  |  |  |  |
| Solvent atoms | 1205 | |  |  |  |  |  |
| Protein chains, residues | 2, 978 | |  |  |  |  |  |
| Average B-factor of protein atoms (Å^2^) | 17 | |  |  |  |  |  |
| Average B-factor of solvent atoms (Å^2^) | 26 | |  |  |  |  |  |
| rms bond lengths (Å) | 0.010 | |  |  |  |  |  |
| rms bond angles (°)  TLS groups  Molprobity clash score, percentile | 1.00  17  2.24, 99^th^ | |  |  |  |  |  |
| ^a^ Values in parentheses indicate statistics for the highest resolution shell. | | |  | |  | | |
|  | |  |  |  | | |  |

**Data S1.** Sequences of *H. pylori* KatA variants.
